# Improving TCR Gene Therapy for Treatment of Haematological Malignancies

**DOI:** 10.1155/2012/404081

**Published:** 2012-01-26

**Authors:** Emma Nicholson, Sara Ghorashian, Hans Stauss

**Affiliations:** Department of Immunology, Royal Free Hospital, University College London, Hampstead Campus, Rowland Hill Street, London NW3 2QG3, UK

## Abstract

Adoptive immunotherapy using TCR gene modified T cells may allow separation of beneficial Graft versus tumour responses from harmful GvHD. Improvements to this include methods to generate high avidity or high affinity TCR, improvements in vector design and reduction in mispairing. Following adoptive transfer, TCR transduced T cells must be able to survive and persist in vivo to give most effective antitumour responses. Central memory or naive T cells have both been shown to be more effective than effector cells at expanding and persisting in vivo. Lymphodepletion may enhance persistence of transferred T cell populations. TCR gene transfer can be used to redirect CD4 helper T cells, and these could be used in combination with CD8+ tumour specific T cells to provide help for the antitumour response. Antigen specific T regulatory T cells can also be generated by TCR gene transfer and could be used to suppress unwanted alloresponses.

## 1. Introduction

Allogeneic haematopoietic stem cell transplantation (HSCT) is an effective treatment for many haematological malignancies. In addition, unselected donor lymphocyte infusions (DLIs) can be utilized to successfully treat relapsed leukaemia after HSCT [1]. Depending on the degree of HLA mismatch, donor T cells recognize alloantigens derived from allogeneic MHC or from polymorphic minor histocompatibility antigens (mHags) expressed by the host. Whilst able to deliver beneficial Graft versus Tumour effects (GvT), alloreactive T cells may also direct their response against normal tissues resulting in Graft versus Host Disease (GvHD), and this is one of the leading causes of transplant-related morbidity and mortality. The incidence of GvHD can be reduced by utilizing T-cell-depleted transplants-but this also leads to an increase in disease relapse rate [2–4]. How best to deliver effective GvT responses whilst minimizing harmful GvHD remains a significant challenge.

Refining the concept of donor lymphocyte infusions by isolating donor lymphocytes that have known tumour reactivity may result in more effective GvT. Falkenburg et al. have utilized donor-derived leukaemia reactive cytotoxic T lymphocytes (CTLs) to treat a patient with relapsed accelerated phase CML after HSCT [5]. The patient, who had previously been resistant to DLI, went on to achieve a complete remission as a result of this therapy. A phase I/II study looking at generating leukaemia reactive CTLs for patients with relapsed leukaemia after HSCT found that whilst this strategy was feasible, it was complex and time consuming requiring improvements before becoming a definitive treatment strategy [6].

As an alternative to isolating tumour reactive lymphocytes, the specificity of T cells can be redirected by retroviral gene transfer of T-cell receptor (TCR) genes. This allows T cells to be generated that are specific for a defined tumour antigen presented by MHC. TCR gene transfer using retroviruses was first demonstrated by Clay et al. who transduced human T cells with a TCR that was specific for a melanoma antigen presented by HLA-A2. These redirected T cells had demonstrable antitumour reactivity in vitro [7]. TCRs targeting a variety of tumour associated antigens (TAA) have now been used for retroviral transduction of T lymphocytes and shown to respond to specific tumour antigens in vitro or provide tumour protection in vivo in murine models post adoptive transfer [8–12].

The first clinical trial using TCR gene modified T cells was for treatment of patients with metastatic melanoma. In this study, autologous peripheral blood lymphocytes were transduced with alpha and beta chains specific for a melanoma TAA, MART-1, and adoptively transferred to a cohort of patients with metastatic melanoma. This resulted in an objective response rate in 2/15 patients (13%) with both responders obtaining long-term disease remission [13]. A high-avidity TCR targeting the same MART-1 epitope has been developed, and this has led to anti tumour responses in 6/20 (30%) of patients treated. In the same trial, a high avidity TCR targeting the gp100 melanoma antigen resulted in tumour regression in 3/16 patients (19%) [14]. TCR-transduced autologous T cells targeting the cancer testis antigen, NYESO1, have been used to treat patients with metastatic melanoma and advanced synovial cell carcinoma resulting in response rates of 45% and 67%, respectively [15]. Despite this, the response rate is still far below that which has been achieved using antigen-specific tumour infiltrating lymphocytes (TILs). Currently the best objective response rate described using autologous TIL in patients with metastatic melanoma is 72%, with 16% achieving complete remission [16]. Clinical trials utilizing TCR-transduced cells for treatment of haematological malignancies are still awaited, but this may allow the delivery of effective GvT responses without harmful GvHD effects.

## 2. Generating High-Affinity and High-Avidity TCR-Transduced T Cells and Reducing Mispairing

Improving the therapeutic GvT effect may be achieved by an increase in the functional avidity of the transduced T cell for its specific tumour antigen. Increasing T cell avidity has been correlated with improved elimination of tumour cells in vivo [17]. Avidity of the TCR is a function of the level of expression of the TCR on the T-cell surface and the individual affinity of the TCR for its cognate peptide-MHC (pMHC). High-avidity CTLs specific for TAA may be absent from the peripheral T cell repertoire as a result of deletion during thymic selection or tolerance induction following encounter of TAA in the periphery.

One method to generate high-avidity TAA-specific CTLs is to immunize human HLA expressing transgenic mice with human TAA peptides thus eliciting an immune response against the human peptide. CTLs that express high-avidity TCRs can then be selected, and the TCR genes isolated and used to transduce peripheral human lymphocytes [18, 19]. Alternatively the allorestricted technique can be utilized: T cell tolerance is self MHC restricted, but peptides presented in the context of an allogeneic MHC molecule will be able to stimulate high-avidity CTLs. TAA peptides presented by MHC molecules of a known HLA haplotype can be used to stimulate lymphocytes in vitro from a HLA-mismatched donor, and, from this, antigen specific high avidity T cells can be isolated [20, 21].

Naturally occurring TCRs are typically of low affinity, normally within the range of 1–100 *μ*mol [22, 23]. TCRs can be modified to increase individual TCR affinity by introducing amino-acid substitutions into the CDR regions of the alpha and beta chains, in particular into CDR3 regions that bind to peptide. TCRs can be selected for increased affinity using yeast or bacteriophage display techniques [24–26]. This has led to the generation of high-affinity TCR which have affinity of up to 1 million times higher than that of wild-type TCR but still retain the ability to recognize specific peptide MHC. Some of the high affinity TCRs do lose antigen specificity as the affinity for pMHC increases, particularly if the TCR is expressed in CD8+ T cells [25]. Zhao et al. transduced CD8+ and CD4+ T cells with a class I-restricted TCR specific for NY-ESO. They found CD8+ cells transduced with high-affinity TCR lost their antigen specificity at very high ranges of affinity whilst CD4+ T cells transduced with high-affinity TCR had a marked increase in reactivity against specific pMHC and tumour cell lines with no evidence of cross-reactivity [27]. One other possible drawback of increasing TCR affinity is that there may be a threshold of affinity above which TCR function begins to plateau or even decrease. Higher-affinity TCRs have a longer dissociation time from pMHC, reducing the number of TCRs bound by any given pMHC which may lead to a reduction in T-cell activation, particularly if antigen is present at low concentration. Thomas et al. have recently demonstrated that TCR with affinity above the normal range trigger faster effector responses than wild-type TCR, but this is associated with a progressive loss of response to low-density antigen as affinity increased [28].

Higher surface expression of TCR has been shown to correlate with an increased responsiveness to specific antigen [29–31]. Improvements in vector design have led to an increased transduction efficiency of the introduced alpha and beta chains leading to an increased surface expression of the TCR. Commonly the TCR genes are inserted into a retroviral cassette although the use of lentiviral vectors has increased. The use of an IRES to link the alpha and beta chains allows transcription of a single mRNA initiating at the long terminal repeat (LTR) of the virus. The upstream gene is translated from the 5′ cap and the downstream gene translated from the IRES element. However this can lead to differential expression of the two genes with the downstream gene being expressed at a lower level [32]. More commonly genes are now being linked by viral derived 2A oligopeptide linker sequences [33]. 2A sequences can be inserted between 2 genes within the one vector backbone. The upstream gene is translated fully, but, upon reaching the 2A sequence, an interaction between the 2A and the ribosome terminates translation. The first protein is released with the 2A sequence fused to its C terminus and the ribosome “skips” to the downstream gene and resumes translation. This can lead to the production of multiply expressed genes within one vector cassette [34]. Direct comparison of retroviral vectors expressing alpha and beta chains linked by IRES or 2A sequences demonstrates that the 2A linker sequences result in higher expression of TCR genes and improved T-cell function [35]. Codon optimization of the insert by replacing rarely used codons with those in more frequent use has also been shown to increase expression level of introduced TCR which leads to an increase in tumour protection in vivo [31].

When introducing a new alpha and beta chain into a T cell, ideally they will pair only with one another. However, the introduced alpha and beta chains may form mixed heterodimers whereby the introduced alpha chain pairs with the endogenous beta chain and the introduced beta chain pairs with the endogenous alpha chain. This can lead to the expression of 2 new TCR specificities by the T cell with the potential to target normal tissues as they will not have undergone selection in the thymus. In addition, this will lead to lower expression of the correctly paired TCR and thus lower the functional avidity of the T cell. There are now a number of methods to reduce mispairing. The introduction of a new disulphide bond into the constant region of the introduced alpha and beta chain ensures that these chains will more efficiently bond with each other and not with the endogenous TCR chains. This has been shown to reduce mispairing and improve the functional avidity of T cells [36–38]. Replacing the constant region of human TCRs with the murine constant region also leads to preferential pairing of the modified alpha and beta chains leading to increased expression of the TCR. As well as preventing mispairing, it has been shown that murine constant region pairs more efficiently with human CD3 resulting in more efficient competition for binding to CD3 [37]. The combination of murinization of constant regions and an additional disulfide bond has an additive effect on reducing mispairing and increasing surface expression of the introduced TCR although neither completely eliminates mispairing [36]. Expression of the introduced TCR may also be increased by suppression of the expression of the endogenous TCR. This has been achieved by transducing human PBMC with a vector which encodes a tumour-specific TCR and a small interfering (siRNA) which specifically downregulates the endogenous TCR [39].

TCR gene transfer experiments have demonstrated that TCR cell surface composition following transduction is dependent on properties of both the introduced TCR and the existing endogenous TCR. This has been described as TCR strength, with “strong” TCRs being more efficiently expressed on the cell surface compared to “weak” TCRs. T-cell strength may be controlled by the intrinsic pairing properties of the alpha and beta chains and also by their ability to bind to CD3 [40]. The introduced TCR must compete with the endogenous TCR prior to expression on the T-cell surface for binding to CD3. TCR and CD3 assemble into a TCR-CD3 complex within the endoplasmic reticulum and once fully assembled, exported to the cell surface. The amount of CD3 within the T cell thus is rate limiting for expression of the introduced TCR. TCR and CD3 can be cotransduced in order to provide an excess of CD3 and thus increase expression of the introduced TCR. Recent data has suggested that the increased surface expression of TCR following cotransduction of CD3 and TCR leads to an increase in tumour protection in vivo (Ahmadi, Blood, in press).

## 3. Improving Survival and Persistence of TCR-Transduced T Cells Following Adoptive Transfer

The ability of transduced T cells to persist long-term, mount robust memory responses and migrate to the site of response following adoptive transfer is key to successful antitumour responses. Persistence of transferred tumour reactive T cells has been shown to correlate with an effective therapeutic effect in adoptive immunotherapy utilizing TIL for treatment of metastatic melanoma [41]. From the earliest trials of adoptive cellular therapy, it has been demonstrated that “younger” TIL- that is, those subjected to fewer rounds of stimulation delivered better antitumour responses. In mouse models, tumour-specific CD8+ T cells that had undergone a number of in vitro stimulations and acquired effector functions were less effective at mediating tumour regression after adoptive transfer [42, 43]. Cells that had a prolonged period in culture acquired a T effector cell phenotype with decreased expression of cell surface markers associated with trafficking to secondary lymphoid organs, for example, CCR7 and CD62L and also downregulation of costimulatory receptors such as CD27 and CD28 [44]. Shorter telomeres were also associated with a reduction in clinical response and reduced in vivo persistence [45]. It is becoming increasingly apparent that the transfer of end stage effector CD8 T cells may not be desirable for adoptive immunotherapy but that transfer of naive or memory cell subsets may be more effective.

Naive, central memory (CM) and effector memory (EM) populations each have distinct phenotypic and functional characteristics. In vitro stimulation of these subsets induces their proliferation and differentiation into cytolytic effector cells. EM T cells preferentially home to peripheral tissues, have immediate effector function on antigen rechallenge but show only poor numeric expansion. They have downregulated CD62L although maintain expression of CCR7. CM T cells have high expression of CD62L and CCR7 and thus can home to and recirculate through secondary lymphoid organs where they can interact efficiently with antigen-presenting cells. They have enhanced proliferative capacity upon antigen reencounter [43, 46]. A comparison of transfer of tumour-specific CM T cells and EM T cells in a murine model of melanoma showed that CM T cells had more efficient in vivo recall responses compared with EM T cells which were associated with superior antitumour ability [43].

Berger et al. looked at the persistence of EM T cells versus CM T cells following adoptive transfer of CD8+ CMV-specific effector cells into unconditioned primates. The effector T cells that were derived from EM T cells only persisted in the blood for a short period and were unable to persist within lymph node or bone marrow or peripheral tissues. They were also unable to differentiate back into an EM T-cell phenotype. In contrast the effector T cells derived from CM T-cells were able to persist in blood and could migrate to bone marrow and lymph nodes and had the ability to differentiate to both CM and EM T cell phenotype. Effector cells derived from CM T cells appeared to be more plastic than EM T cells, retaining the ability to revert to both a CD62L− and CD62+ memory cell phenotype [47].

However, this study did not address the role of naive T cells for use in adoptive immunotherapy. Hinrichs et al. utilizing a murine adoptive transfer model have suggested that naive T cells may be superior to T CM cells. T cells were isolated from pmel-1 TCR-transgenic mice which express TCR specific for self-antigen, gp100 that is also expressed by murine melanoma cells. Sublethally irradiated mice with established B16 melanoma tumours received adoptive transfer of CD8+ naive T cells (CD62L+ CD44low) or CM T cells (CD62L+CD44high). All mice also received specific peptide vaccination and IL-2. Both naive and CM T cells were able to mediate tumour regression following adoptive transfer, but effector cells derived from naive T cells had superior antitumour activity. Adoptively transferred naive T cells also showed greater expansion within spleen and draining LN and also produced higher amounts of IFN-*γ* and IL-2 following ex vivo restimulation [48].

A novel way to try to prevent T cells acquiring a T effector phenotype prior to adoptive transfer is to target the Wnt/Beta Catenin pathway which is thought to be important in the generation and maintenance of T-cell memory [49]. The Wnt/beta catenin pathway can be activated artificially in vitro and in vivo utilizing inhibitors of glycogen synthase kinase 3*β*(Gsk-3*β*). Gsk-3*β* inhibitors lead to an accumulation of beta catenin which mimics Wnt signaling [50]. Utilizing pmel-1 transgenic T cells, Gattonini et al. primed naive T cells in the presence of Gsk-3*β* inhibitors. This inhibited cells from acquiring a T effector phenotype and also generated a CD44 low CD62L high T-cell population which had properties of stem cells, that is, robust self-renewal and the capacity to generate CM, EM, and effector T cells. They also had a characteristic phenotype, with high expression of Sca1, CD122, and Bcl2, previously described by Zhang et al. as CD8 memory stem cells [51]. Following adoptive transfer in a tumour model they had superior proliferative potential and superior antitumour function when compared to CM T cells or EM T cells [50]. Targeting of Wnt/beta catenin signaling pathways to generate CD8 memory stem cells in combination with TCR transduced T cells represents an attractive strategy for adoptive immunotherapy.

Lymphodepletion as a result of conditioning therapy preadoptive transfer has been shown to increase persistence of transferred T cells. Trials in melanoma patients conditioned with chemotherapy or chemotherapy plus total body irradiation demonstrated that inducing lymphopaenia improved the persistence of the transferred T-cell population [16, 41, 52]. Increasing the level of lymphodepletion in mouse models also demonstrated an increase in persistence of T cells and improvement in antitumour effect [53]. Lymphodepletion results in a spontaneous homeostatic proliferation of the remaining peripheral T cells which can lead to reconstitution of the peripheral lymphocyte pool [54, 55]. In addition T cells that are adoptively transferred into lymphopaenic hosts undergo the same homeostatic proliferation [56]. This process is dependent on homeostatic cytokines, IL-7 and IL-15, and also interaction between TCR and self-peptide/MHC and is similar to normal homeostatic mechanisms that maintain the peripheral T-cell repertoire [22, 57–60]. The degree of expansion of T cells following adoptive transfer is likely to be controlled by access to IL-7 and IL-15 and also the availability of self-peptide/MHC ligands. T cells which have a higher affinity for self-peptide/MHC may have a selective advantage in the lymphopaenic environment and undergo more rapid and efficient expansion [61–63]. This may benefit TCR-transduced T cells specific for tumour-associated antigens which are self-antigens, leading to increased proliferation and persistence following adoptive transfer. It may also be possible to augment this proliferation by increased exposure to specific antigen which could be achieved by peptide vaccination at the time of adoptive transfer which may also give transferred cells an advantage over non-antigen-specific residual host T cells. [64]. It has been shown that CD4+ CD25+ FoxP3+ T regulatory cells also undergo homeostatic proliferation and thus may act to control cell expansion in the lymphopaenic environment [51, 65].

## 4. The Role of TCR-Transduced CD4+ T Cells in the Treatment of Haematological Malignancies

**Figure 1:**
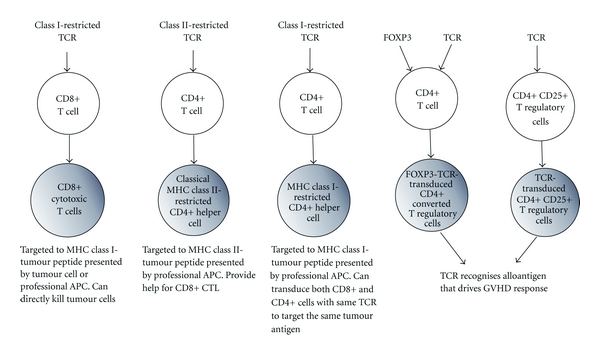
Populations of antigen specific T cells that can be generated by TCR gene transfer for use in adoptive immunotherapy. Retroviral gene transfer can be used to generate different populations of T cells for use in adoptive immunotherapy in the setting of haematological malignancy. Both CD8 and CD4 T cells can be transduced with class I-restricted TCR of the same specificity, targeting the same tumour antigen. Alternatively CD4 T cells can be transduced with class II-restricted TCR-specific tumour antigen presented by class II MHC. Antigen specific T regulatory cells can be generated by TCR transduction of CD4+ CD25+ T regulatory cells or CD4+ T cells can be cotransduced with TCR and FOXP3 resulting in production of antigen-specific converted CD4+ T regulatory cells. Antigen-specific T regulatory cells could be adoptively transferred in the context of HSCT or DLI to reduce harmful GvHD responses of T effector cells.

Most adoptive immunotherapy protocols have focused on the transfer of CD8+ tumour-specific T cells. However, in the absence of CD4 help, CD8+ T cells have impaired function, persistence, and cannot mount effective memory responses [66–68]. The generation of both TCR-transduced CD8+ and CD4+ T helper cells may have a beneficial effect on sustained tumour control (Figure 1).

Quezada et al. explored the role of classical class II-restricted CD4+ T cells in a murine model of advanced melanoma. Adoptive transfer of small numbers of naive class II-restricted tumour specific CD4+ T cells into irradiated recipients led to in vivo differentiation and expansion of CD4+ T cells and tumour regression. These CD4+ T cells acquired cytotoxic activity and directly rejected the tumour in a class II-dependent manner. The efficacy of CD4+ T cells was increased by concurrent blockade of CTLA-4 resulting in greater expansion of CD4+ T cells and decreased numbers of T regulatory cells [69].

The use of CD4 + T cells for adoptive immunotherapy is limited due to a lack of well-characterized tumour antigens presented by class II MHC. In addition the majority of tumour cells are class II negative and therefore cannot directly present antigen to CD4+ T cells. To overcome these limitations, CD4+ T cells expressing class I restricted TCR have been generated by gene transfer. It has been demonstrated that CD4 T cells transduced with class I-restricted TCR can provide antigen-specific helper functions. Some class I-restricted TCRs are independent of the CD8 coreceptor and function in CD4+ T cells in the absence of additional CD8 [19]. However the majority of class I-restricted TCRs are CD8 dependent and thus require cotransduction of CD8 to be fully functional in CD4+ T cells. CD8 stabilizes the interaction between the TCR and peptide-MHC and increases the duration of this interaction. Kessels et al. transduced CD4+ T cells with the class I-restricted OT1 TCR [70]. OT1 TCR CD4+ T cells required cotransduction of the alpha and beta chains of CD8 in order to respond to specific antigen, and the transduction of just the alpha chain of CD8 was not sufficient to provide full CD8 coreceptor function. Morris et al. transduced CD4+ T cells with a class I-restricted TCR which is specific for NP peptide and is partially CD8 dependent [9]. CD4+ TCR-transduced T+ cells were able to produce IL-2 and proliferate in vitro in response to class II-negative tumour cells expressing specific peptide, but these cells were not able to generate an IFN-*γ* response. In vivo, CD4+ T cells could provide help for CTL-mediated tumour eradication, and these cells persisted in vivo for up to 90 days after tumour regression and were able to reexpand following tumour challenge. The generation of class I-restricted TCRs is a promising strategy with the advantage that tumour-specific CD4+ and CD8+ T cells targeting the same epitope can be generated.

CD4+ CD25+ FOXP 3+ T regulatory cells (CD4+ T regs) play a crucial role in the control of GvHD. This has been demonstrated via the depletion of CD4+ T regs from donor cells in murine MHC-mismatched transplant models. These transplants were done using a variety of different strain combinations, and the depletion of CD4+ T regs resulted in an increase in GVHD and an increase in mortality [71]. Adoptive transfer of additional CD4+ T regs with T effector cells is one possible strategy of reducing GvHD burden after allogeneic transplant. The infusion of freshly isolated polyclonal CD4+ T regs in a 1 : 1 ratio with T effector cells has been shown to lead to a delay in development of GvHD in a MHC-mismatched murine transplant [72, 73]. Polyclonal CD4+ T regs can be expanded dramatically ex vivo using high-dose IL-2 and anti-CD3/CD28 beads or can also be expanded ex vivo utilizing irradiated allogeneic splenocytes prior to adoptive transfer. Trenado et al. cultured CD4+T regs in the presence of allogeneic irradiated splenocytes which led to a 1000-fold expansion. These alloreactive CD4+ T regs were infused to lethally irradiated mice in a 1 : 1 ratio with T effector cells in a murine MHC-mismatched transplant model. This led to complete protection from GvHD. CD4+ T regs that were expanded using third-party splenocytes were of much lower efficacy than alloreactive CD4+ T regs. They also demonstrated that the reduction in GvHD did not lead to reduction in GvT responses mediated by CD8+ effector T cells following the injection of A20 leukaemia cell lines to recipient mice on the day of transplant [74]. Other groups have also demonstrated the adoptive transfer of murine CD4+ T regs in addition to HSCT can reduce GVHD whilst maintaining GVT effects [75, 76].

The use of antigen-specific CD4+ T regs is more effective at reducing GvHD than a polyclonal population [77]. CD4+ T regs require to meet specific antigen in order to be activated although once activated can mediate suppression of T effector cells regardless of specificity. However, if CD4+ T regs are antigen specific, this has the advantage that they can be targeted to the same antigen as the T effector cell, targeting the suppressive response directly to the site of the harmful effector response. It has been shown that the recruitment of T regulatory cells to the site of GvHD is critical for the suppression of alloreactive T cells [78].

Gene transfer of antigen-specific CD4+ T cells with FOXP3 is one possible mechanism of generating large numbers of antigen-specific CD4+ T regs. The ectopic expression of FOXP3 had been shown to convert CD4+ T cells to regulatory T cells that are phenotypically and functionally the same as CD4+ T regs. Albert et al. produced CD4+ T regs by transducing CD4+ CD25- TCR-transgenic cells with FOXP3 and found that FOXP3-transduced T regs were as effective as wild-type CD4+ T regs at suppressing GvHD [77]. Antigen-specific CD4+ T regs may also be generated by the transduction of a specific TCR into CD4+ T regs or also by the cotransduction of both TCR and FOXP3 into conventional CD4+ T cells. In a mouse model of inflammatory arthritis, both TCR-transduced T regulatory cells and FOXP3 and TCR-co-transduced CD4+ T cells were able to mediate suppressive effects following adoptive transfer leading to reduction in progression of inflammatory arthritis [79]. There is some concern that CD4+ T regs may convert following adoptive transfer to TH17 cells thus abrogating their suppressive function [80]. However FOXP3 and TCR-co-transduced CD4+ T cells should be a more stable population due to the persistent and stable expression of FOXP3 from a strong retroviral promoter.

## 5. Tumour Antigen Selection

In order to develop effective adoptive immunotherapy protocols, it is important that the correct TAA or epitope is targeted. The use of high throughput screening of cancer genomes can identify large numbers of novel TAA, and it is important that research is focused, leading to more effective translation of TCR gene therapy and vaccine strategies into clinical practice. An ideal tumour antigen would be one that is widely expressed in different tumour types and not unique to individual patients. Targeting tumour antigens that are essential to the oncogenic process or cancer cell survival may induce sustained tumour regression. Specifically targeting a single epitope, however, may lead to the selection of tumour variants that lack the target antigen as a result of antigen loss or aberrant presentation via MHC loss [81–83]. Selecting a target that does not lead to autoimmune damage will also be of paramount importance. This has been recently demonstrated in a study where lymphocytes transduced with a high-avidity TCR specific for carcinoembryonic antigen (CEA) were administered to 3 patients with metastatic colorectal cancer. All patients in the study developed severe inflammatory colitis as a result of the TCR gene-modified T cells recognizing CEA expressed within normal colonic epithelium [84].

A National Cancer Institute Pilot project has been developed to try to prioritize antigens for further research and development [85]. This utilized a methodology termed Analytic Hierarchy Process (AHP), which breaks down complex problems into a hierarchy of subproblems allowing comparison on a pairwise basis. 75 cancer antigens were ranked and scored based on criteria that had been defined and given differential weightings by a panel of experts. The criteria used for ranking (and in order of weighting) were demonstrable therapeutic function of antigen, known immunogenicity, oncogenic function, whether antigen is expressed widely or uniquely by tumour cells, expression level of antigen within tumours, expression by tumour stem cells, the number of patients with antigen positive cells, the number of epitopes of the tumour antigen and whether it is able to bind to multiple MHC molecule, and finally the cellular location of the antigen. The three top ranked antigens were WT1, MUC1, and LMP2. The antigens that have already been extensively researched score higher as there is more supportive evidence of therapeutic function and immunogenicity. For some of the subcriteria, there exists only limited information, but this process is dynamic, allowing reanalysis as further evidence accrues.

## 6. Safety Considerations of TCR-Transduced T Cells

In addition to reducing the efficacy of TCR transduced T cells, mispairing is also a concern with regards to safety of TCR-transferred T cells for clinical use. Bendle et al. have demonstrated that TCR-transduced T cells can induce GVHD following adoptive transfer. This is not a result of the on-target toxicity of the introduced TCR but is a result of the formation of new heterodimers that have off-target toxicity directed against normal tissues [86]. This stresses the importance of utilizing mechanisms to prevent mispairing of the introduced alpha and beta chains.

Lymphocytes can be transduced with suicide genes which enables selective elimination of transduced cells should GvHD develop following adoptive transfer. The most extensively studied to date is herpes simplex thymidine kinase (HSV-TK). HSV-TK phosphorylates ganciclovir to a toxic metabolite which interrupts DNA elongation resulting in the selective death of cells transduced with HSV-TK following the infusion of ganciclovir. When administered to patients with relapsed haematological malignancy after allogeneic HLA-matched HSCT, rates of GvT using HSV-TK-transduced lymphocytes were comparable to that of DLI using unmanipulated lymphocytes. The administration of ganciclovir for treatment of GVHD or for CMV reactivation selectively eliminated HSV-TK-transduced cells and improved GvHD [87]. HSV-TK-transduced lymphocytes have also been investigated as a coinfusion at the time of T-depleted HLA identical HSCT and also as incremental add back infusion following haploidentical transplant [88, 89]. Although a promising strategy for control of GVHD after DLI, the efficacy may be limited due to the immunogenicity of HSV-TK leading to a decrease in persistence of transduced cells [90]. In addition the administration of ganciclovir for treatment of CMV reactivation results in the premature destruction of HSV-TK-transduced cells leading to loss of efficacy.

Another suicide gene strategy in development is based on human apoptosis proteins. A fusion protein consisting of a late stage apoptosis molecule, caspase 9 fused to a FK506-binding protein (FK506BP) analogue has been transduced into lymphocytes. Apoptosis of the transduced cells is induced following the administration of a chemical inducer of dimerization (CID) which results in aggregation and activation of caspase 9. The CID which has been used is a nontoxic FK506 analog which has been modified so that it has much higher affinity for the modified FK506BP than the endogenous FK506BP. A single dose of CID resulted in death of >99% of transduced cells both in vitro and in vivo. These molecules are human derived and therefore should in theory be less immunogenic than HSV-TK [91].

 An additional safety concern of gene therapy is insertional mutagenesis secondary to gene insertion into the host chromosome leading to disruption or activation of cellular genes. Out of the 19 X-linked severe combined immunodeficiency patients treated with retrovirally transduced haematopoietic stem cells there have been 5 cases of T-cell leukaemia reported [92–94]. In 4 of the 5 cases, the leukaemia arose secondary to retroviral integration in the region of a T-cell oncogene, LMO2, resulting in its deregulated expression. In these clinical trials, the vector expressed the IL-2 receptor gamma (IL-2R-*γ*) chain, and there is some controversy over whether this gene itself had a role as a cooperating oncogene. Insertions near both the LM02 and IL-2R-*γ* chain gene have been described in murine T-cell leukaemia [95]. In addition, adoptive transfer of HSC transduced with lentiviral vectors expressing very high levels of the IL-2R-*γ* chain led to a high incidence of lymphoma in a murine model system [96]. Pike-Overzet et al., however, have shown that the overexpression of IL-2R-*γ* chain did not have any effect on haematopoietic cell development whilst overexpression of LMO2 led to a block in T-cell development leading to a preleukaemic phenotype [97]. Modlich et al. also found that vector-mediated expression of IL-2R-*γ* chain was not sufficient to lead to induction of leukaemia following HSCT in mouse models using both lentiviral and retroviral vectors [98].

**All cases of leukaemia arising secondary to insertional mutagenesis have involved the use of retroviral vectors which have LTRs with strong enhancer/promoters elements. Strong LTRs may cause the transcription of cellular genes in addition to the transgene which can lead to unregulated gene expression. By using self inactivating vectors (SIN), it may be possible to reduce the risk of insertional mutagenesis. SIN vectors contain a deletion within the U3 region of the 3′LTR which leads to inactivation of the 5′LTR following reverse transcription, thus, inactivating both LTRs [99, 100]. Any expression of the transgene is then driven by an internal promoter. The use of SIN in preclinical models suggests that they have a lower oncogenic potential [101–103].

Both lentiviral and gamma retroviral vectors integrate in a semirandom fashion with a bias towards insertion into transcriptional units. Gamma retroviral vectors tend to integrate close to transcription start sites or DNA regulatory areas such as CpG islands. Insertions of viral LTR at these points will have a higher probability of causing aberrant gene expression [104, 105]. In contrast, lentiviral vectors tend to integrate within active transcription units and as a result may have a safer insertion profile [106, 107]. Cattoglio et al. analyzed gammaretroviral and lentiviral vector insertion “hot spots” in human cord blood and bone marrow derived CD34+ HSC which had been transduced in vitro. Gamma retroviral vector integration (but not lentiviral) occurred at high frequency (i.e., >20%) in “hot spot” areas enriched in protooncogenes and genes involved in control of cell proliferation [108]. Using a tumour prone mouse model, Montini et al. have compared the oncogenic potential of lentiviral and gamma retroviral vectors, both of which had equivalent strength LTRs and found that lentiviral vectors required a 10-fold higher integration load than retroviral vectors to induce oncogenesis [101]. Only lentiviral vectors that had an active LTR led to insertional transformation of HSCs whereas SIN lentiviral vectors did not lead to transformation above background levels.

The risk of insertional mutagenesis may also be affected by the cell type being transduced, and there is a lower risk of transformation by transducing terminally differentiated cells as opposed to HSC. A study looking at long-term followup of patients receiving T cells transduced with thymidine kinase genes in the context of HSCT demonstrated that up to 20% of gene insertions resulted in altered gene expression of neighbouring genes, but there had been no incidence of clonal selection in these cells [109]. To date, there have been no cases of leukaemia or malignancy arising as a result of adoptive transfer of gene-modified T lymphocytes.

## 7. Conclusion

TCR gene transfer to produce antigen-specific T cells represents a targeted approach to treatment of haematological malignancies which may generate more specific GvT responses whilst reducing harmful GvHD responses. In addition to using TCR-transduced T cells to enhance GvT, we can also generate antigen-specific T regs to directly suppress harmful GVHD responses. Experience from clinical trials of melanoma antigen-specific TCR-transduced T cells has highlighted areas where this technique can be further refined and improved. Improvements in vector design, generation of high-avidity TCR, and reduction of TCR mispairing has all led to improvements in preclinical models. The importance of phenotype and subtype of the transferred population and the host environment into which they are transferred is becoming increasingly evident. Clinical trials utilizing WT1 TCR-transduced lymphocytes for treatment of haematological malignancies are due to commence in the near future hopefully leading to progress translating this technique into wider clinical use.

## References

[1] Kolb HJ, Schattenberg A, Goldman JM (1995). Graft-versus-leukemia effect of donor lymphocyte transfusions in marrow grafted patients. *Blood*.

[2] Marmont AM, Horowitz MM, Gale RP (1991). T-cell depletion of HLA-identical transplants in leukemia. *Blood*.

[3] Horowitz MM, Gale RP, Sondel PM (1990). Graft-versus-leukemia reactions after bone marrow transplantation. *Blood*.

[4] Goldman JM, Gale RP, Horowitz MM (1988). Bone marrow transplantation for chronic myelogenous leukemia in chronic phase. Increased risk for relapse associated with T-cell depletion. *Annals of Internal Medicine*.

[5] Falkenburg JHF, Wafelman AR, Joosten P (1999). Complete remission of accelerated phase chronic myeloid leukemia by treatment with leukemia-reactive cytotoxic T lymphocytes. *Blood*.

[6] Marijt E, Wafelman A, Van Der Hoorn M (2007). Phase I/II feasibility study evaluating the generation of leukemia-reactive cytotoxic T lymphocyte lines for treatment of patients with relapsed leukemia after allogeneic stem cell transplantation. *Haematologica*.

[7] Clay TM, Custer MC, Sachs J, Hwu P, Rosenberg SA, Nishimura MI (1999). Efficient transfer of a tumor antigen-reactive TCR to human peripheral blood lymphocytes confers anti-tumor reactivity. *Journal of Immunology*.

[8] Xue SA, Gao L, Hart D (2005). Elimination of human leukemia cells in NOD/SCID mice by WT1-TCR gene-transduced human T cells. *Blood*.

[9] Morris EC, Tsallios A, Bendle GM, Xue SA, Stauss HJ (2005). A critical role of T cell antigen receptor-transduced MHC class I-restricted helper T cells in tumor protection. *Proceedings of the National Academy of Sciences of the United States of America*.

[10] Zhao Y, Zheng Z, Robbins PF, Khong HT, Rosenberg SA, Morgan RA (2005). Primary human lymphocytes transduced with NY-ESO-1 antigen-specific TCR genes recognize and kill diverse human tumor cell lines. *Journal of Immunology*.

[11] Hughes MS, Yu YYL, Dudley ME (2005). Transfer of a TCR gene derived from a patient with a marked antitumor response conveys highly active T-cell effector functions. *Human Gene Therapy*.

[12] Heemskerk MHM, Hoogeboom M, Hagedoorn R, Kester MGD, Willemze R, Falkenburg JHF (2004). Reprogramming of virus-specific T cells into leukemia-reactive T cells using T cell receptor gene transfer. *Journal of Experimental Medicine*.

[13] Morgan RA, Dudley ME, Wunderlich JR (2006). Cancer regression in patients after transfer of genetically engineered lymphocytes. *Science*.

[14] Johnson LA, Morgan RA, Dudley ME (2009). Gene therapy with human and mouse T-cell receptors mediates cancer regression and targets normal tissues expressing cognate antigen. *Blood*.

[15] Robbins PF, Morgan RA, Feldman SA (2011). Tumor regression in patients with metastatic synovial cell sarcoma and melanoma using genetically engineered lymphocytes reactive with NY-ESO-1. *Journal of Clinical Oncology*.

[16] Dudley ME, Yang JC, Sherry R (2008). Adoptive cell therapy for patients with metastatic melanoma: evaluation of intensive myeloablative chemoradiation preparative regimens. *Journal of Clinical Oncology*.

[17] Alexander-Miller MA, Leggatt GR, Berzofsky JA (1996). Selective expansion of high- or low-avidity cytotoxic T lymphocytes and efficacy for adoptive immunotherapy. *Proceedings of the National Academy of Sciences of the United States of America*.

[18] Stanislawski T, Voss RH, Lotz C (2001). Circumventing tolerance to a human MDM2-derived tumor antigen by TCR gene transfer. *Nature Immunology*.

[19] Kuball J, Schmitz FW, Voss RH (2005). Cooperation of human tumor-reactive CD4+ and CD8+ T cells after redirection of their specificity by a high-affinity p53A2.1-specific TCR. *Immunity*.

[20] Sadovnikova E, Stauss HJ (1996). Peptide-specific cytotoxic T lymphocytes restricted by nonself major histocompatibility complex class I molecules: reagents for tumor immunotherapy. *Proceedings of the National Academy of Sciences of the United States of America*.

[21] Sadovnikova E, Jopling LA, Soo KS, Stauss HJ (1998). Generation of human tumor-reactive cytotoxic T cells against peptides presented by non-self HLA class I molecules. *European Journal of Immunology*.

[22] Goldrath AW, Bevan MJ (1999). Selecting and maintaining a diverse T-cell repertoire. *Nature*.

[23] Ashton-Rickardt PG, Bandeira A, Delaney JR (1994). Evidence for a differential avidity model of T cell selection in the thymus. *Cell*.

[24] Li Y, Moysey R, Molloy PE (2005). Directed evolution of human T-cell receptors with picomolar affinities by phage display. *Nature Biotechnology*.

[25] Holler PD, Chlewicki LK, Kranz DM (2003). TCRs with high affinity for foreign pMHC show self-reactivity. *Nature Immunology*.

[26] Holler PD, Lim AR, Cho BK, Rund LA, Kranz DM (2001). CD8-T cell transfectants that express a high affinity T cell receptor exhibit enhanced peptide-dependent activation. *Journal of Experimental Medicine*.

[27] Zhao Y, Bennett AD, Zheng Z (2007). High-affinity TCRs generated by phage display provide CD4+ T cells with the ability to recognize and kill tumor cell lines. *Journal of Immunology*.

[28] Thomas S, Xue S-A, Bangham C, Jakobsen BK, Morris EC, Stauss HJ (2011). Human T cells expressing affinity-matured TCR display accelerated responses but fail to recognize low density of MHC-peptide antigen. *Blood*.

[29] Kim DT, Rothbard JB, Bloom DD, Fathman CG (1996). Quantitative analysis of T cell activation: role of TCR/ligand density and TCR affinity. *Journal of Immunology*.

[30] Jorritsma A, Gomez-Eerland R, Dokter M (2007). Selecting highly affine and well-expressed TCRs for gene therapy of melanoma. *Blood*.

[31] De Witte MA, Jorritsma A, Kaiser A (2008). Requirements for effective antitumor responses of TCR transduced T cells. *Journal of Immunology*.

[32] Mizuguchi H, Xu Z, Ishii-Watabe A, Uchida E, Hayakawa T (2000). IRES-dependent second gene expression is significantly lower than cap-dependent first gene expression in a bicistronic vector. *Molecular Therapy*.

[33] Donnelly MLL, Hughes LE, Luke G (2001). The “cleavage” activities of foot-and-mouth disease virus 2A site-directed mutants and naturally occurring “2A-like” sequences. *Journal of General Virology*.

[34] Szymczak AL, Workman CJ, Wang Y (2004). Correction of multi-gene deficiency in vivo using a single “self-cleaving” 2A peptide-based retroviral vector. *Nature Biotechnology*.

[35] Leisegang M, Engels B, Meyerhuber P (2008). Enhanced functionality of T cell receptor-redirected T cells is defined by the transgene cassette. *Journal of Molecular Medicine*.

[36] Thomas S, Xue SA, Cesco-Gaspere M (2007). Targeting the wilms tumor antigen 1 by TCR gene transfer: TCR variants improve tetramer binding but not the function of gene modified human T cells. *Journal of Immunology*.

[37] Cohen CJ, Li YF, El-Gamil M, Robbins PF, Rosenberg SA, Morgan RA (2007). Enhanced antitumor activity of T cells engineered to express T-cell receptors with a second disulfide bond. *Cancer Research*.

[38] Kuball J, Dossett ML, Wolfl M (2007). Facilitating matched pairing and expression of TCR chains introduced into human T cells. *Blood*.

[39] Okamoto S, Mineno J, Ikeda H (2009). Improved expression and reactivity of transduced tumor-specific TCRs in human lymphocytes by specific silencing of endogenous TCR. *Cancer Research*.

[40] Heemskerk MHM, Hagedoorn RS, Van Der Hoorn MAWG (2007). Efficiency of T-cell receptor expression in dual-specific T cells is controlled by the intrinsic qualities of the TCR chains within the TCR-CD3 complex. *Blood*.

[41] Robbins PF, Dudley ME, Wunderlich J (2004). Cutting edge: persistence of transferred lymphocyte clonotypes correlates with cancer regression in patients receiving cell transfer therapy. *Journal of Immunology*.

[42] Gattinoni L, Klebanoff CA, Palmer DC (2005). Acquisition of full effector function in vitro paradoxically impairs the in vivo antitumor efficacy of adoptively transferred CD8+ T cells. *Journal of Clinical Investigation*.

[43] Klebanoff CA, Gattinoni L, Torabi-Parizi P (2005). Central memory self/tumor-reactive CD8+ T cells confer superior antitumor immunity compared with effector memory T cells. *Proceedings of the National Academy of Sciences of the United States of America*.

[44] Powell DJ, Dudley ME, Robbins PF, Rosenberg SA (2005). Transition of late-stage effector T cells to CD27+ CD28 + tumor-reactive effector memory T cells in humans after adoptive cell transfer therapy. *Blood*.

[45] Shen X, Zhou J, Hathcock KS (2007). Persistence of tumor infiltrating lymphocytes in adoptive immunotherapy correlates with telomere length. *Journal of Immunotherapy*.

[46] Roberts AD, Ely KH, Woodland DL (2005). Differential contributions of central and effector memory T cells to recall responses. *Journal of Experimental Medicine*.

[47] Berger C, Jensen MC, Lansdorp PM, Gough M, Elliott C, Riddell SR (2008). Adoptive transfer of effector CD8+ T cells derived from central memory cells establishes persistent T cell memory in primates. *Journal of Clinical Investigation*.

[48] Hinrichs CS, Borman ZA, Cassard L (2009). Adoptively transferred effector cells derived from naïve rather than central memory CD8+ T cells mediate superior antitumor immunity. *Proceedings of the National Academy of Sciences of the United States of America*.

[49] Gattinoni L, Ji Y, Restifo NP (2010). Wnt/*β*-catenin signaling in T-cell immunity and cancer immunotherapy. *Clinical Cancer Research*.

[50] Gattinoni L, Zhong XS, Palmer DC (2009). Wnt signaling arrests effector T cell differentiation and generates CD8+ memory stem cells. *Nature Medicine*.

[51] Zhang Y, Joe G, Hexner E, Zhu J, Emerson SG (2005). Host-reactive CD8+ memory stem cells in graft-versus-host disease. *Nature Medicine*.

[52] Dudley ME, Wunderlich JR, Yang JC (2005). Adoptive cell transfer therapy following non-myeloablative but lymphodepleting chemotherapy for the treatment of patients with refractory metastatic melanoma. *Journal of Clinical Oncology*.

[53] Wrzesinski C, Paulos CM, Gattinoni L (2007). Hematopoietic stem cells promote the expansion and function of adoptively transferred antitumor CD8+ T cells. *Journal of Clinical Investigation*.

[54] Bell EB, Sparshott SM, Drayson MT, Ford WL (1987). The stable and permanent expansion of functional T lymphocytes in athymic nude rats after a single injection of mature T cells. *Journal of Immunology*.

[55] Rocha B, Dautigny N, Pereira P (1989). Peripheral T lymphocytes: expansion potential and homeostatic regulation of pool sizes and CD4/CD8 ratios in vivo. *European Journal of Immunology*.

[56] Oehen S, Brduscha-Riem K (1999). Naive cytotoxic T lymphocytes spontaneously acquire effector function in lymphocytopenic recipients: a pitfall for T cell memory studies?. *European Journal of Immunology*.

[57] Kieper WC, Jameson SC (1999). Homeostatic expansion and phenotypic conversion of naïve T cells in response to self peptide/MHC ligands. *Proceedings of the National Academy of Sciences of the United States of America*.

[58] Viret C, Wong FS, Janeway CA (1999). Designing and maintaining the mature TCR repertoire: the continuum of self-peptide:self-MHC complex recognition. *Immunity*.

[59] Kondrack RM, Harbertson J, Tan JT, McBreen ME, Surh CD, Bradley LM (2003). Interleukin 7 Regulates the Survival and Generation of Memory CD4 Cells. *Journal of Experimental Medicine*.

[60] Tan JT, Ernst B, Kieper WC, LeRoy E, Sprent J, Surh CD (2002). Interleukin (IL)-15 and IL-7 jointly regulate homeostatic proliferation of memory phenotype CD8+ cells but are not required for memory phenotype CD4+ cells. *Journal of Experimental Medicine*.

[61] Mackall CL, Bare CV, Granger LA, Sharrow SO, Titus JA, Gress RE (1996). Thymic-independent T cell regeneration occurs via antigen-driven expansion of peripheral T cells resulting in a repertoire that is limited in diversity and prone to skewing. *Journal of Immunology*.

[62] Moses CT, Thorstenson KM, Jamesont SC, Khoruts A (2003). Competition for self ligands restrains homeostatic proliferation of naive CD4 T cells. *Proceedings of the National Academy of Sciences of the United States of America*.

[63] Troy AE, Shen H (2003). Cutting edge: homeostatic proliferation of peripheral T lymphocytes is regulated by clonal competition. *Journal of Immunology*.

[64] Wang LX, Li R, Yang G (2005). Interleukin-7-dependent expansion and persistence of melanoma-specific T cells in lymphodepleted mice lead to tumor regression and editing. *Cancer Research*.

[65] Gavin MA, Clarke SR, Negrou E, Gallegos A, Rudensky A (2002). Homeostasis and anergy of CD4+CD25+ suppressor T cells in vivo. *Nature Immunology*.

[66] Sun JC, Bevan MJ (2003). Defective CD8 T cell memory following acute infection without CD4 T cell help. *Science*.

[67] Janssen EM, Lemmens EE, Wolfe T, Christen U, Von Herrath MG, Schoenberger SP (2003). CD4+ T cells are required for secondary expansion and memory in CD8+ T lymphocytes. *Nature*.

[68] Shedlock DJ, Shen H (2003). Requirement for CD4 T cell help in generating functional CD8 T cell memory. *Science*.

[69] Quezada SA, Simpson TR, Peggs KS (2010). Tumor-reactive CD4+ T cells develop cytotoxic activity and eradicate large established melanoma after transfer into lymphopenic hosts. *Journal of Experimental Medicine*.

[70] Kessels HWHG, Schepers K, Van Den Boom MD, Topham DJ, Schumacher TNM (2006). Generation of T cell help through a MHC class I-restricted TCR. *Journal of Immunology*.

[71] Taylor PA, Lees CJ, Blazar BR (2002). The infusion of ex vivo activated and expanded CD4+CD25+ immune regulatory cells inhibits graft-versus-host disease lethality. *Blood*.

[72] Cohen JL, Trenado A, Vasey D, Klatzmann D, Salomon BL (2002). CD4+CD25+ immunoregulatory T cells: new therapeutics for graft-versus-host disease. *Journal of Experimental Medicine*.

[73] Hoffmann P, Ermann J, Edinger M, Garrison Fathman C, Strober S (2002). Donor-type CD4+CD25+ regulatory T cells suppress lethal acute graft-versus-host disease after allogeneic bone marrow transplantation. *Journal of Experimental Medicine*.

[74] Trenado A, Charlotte F, Fisson S (2003). Recipient-type specific CD4+CD25+ regulatory T cells favor immune reconstitution and control graft-versus-host disease while maintaining graft-versus-leukemia. *Journal of Clinical Investigation*.

[75] Edinger M, Hoffmann P, Ermann J (2003). CD4+CD25+ regulatory T cells preserve graft-versus-tumor activity while inhibiting graft-versus-host disease after bone marrow transplantation. *Nature Medicine*.

[76] Jones SC, Murphy GF, Korngold R (2003). Post-hematopoietic cell transplantation control of graft-versus-host disease by donor CD4+25+ T cells to allow an effective graft-versus-leukemia response. *Biology of Blood and Marrow Transplantation*.

[77] Albert MH, Liu Y, Anasetti C, Yu XZ (2005). Antigen-dependent suppression of alloresponses by Foxp3-induced regulatory T cells in transplantation. *European Journal of Immunology*.

[78] Wysocki CA, Panoskaltsis-Mortari A, Blazar BR, Serody JS (2005). Leukocyte migration and graft-versus-host disease. *Blood*.

[79] Wright GP, Notley CA, Xue SA (2009). Adoptive therapy with redirected primary regulatory T cells results in antigen-specific suppression of arthritis. *Proceedings of the National Academy of Sciences of the United States of America*.

[80] Koenen HJPM, Smeets RL, Vink PM, Van Rijssen E, Boots AMH, Joosten I (2008). Human CD25highFoxp3pos regulatory T cells differentiate into IL-17 producing cells. *Blood*.

[81] Khong HT, Restifo NP (2002). Natural selection of tumor variants in the generation of "tumor escape" phenotypes. *Nature Immunology*.

[82] Restifo NP, Esquivel F, Kawakami Y (1993). Identification of human cancers deficient in antigen processing. *Journal of Experimental Medicine*.

[83] Schreiber RD, Old LJ, Smyth MJ (2011). Cancer immunoediting: integrating immunity's roles in cancer suppression and promotion. *Science*.

[84] Parkhurst MR, Yang JC, Langan RC (2011). T cells targeting carcinoembryonic antigen can mediate regression of metastatic colorectal cancer but induce severe transient colitis. *Molecular Therapy*.

[85] Cheever MA, Allison JP, Ferris AS (2009). The prioritization of cancer antigens: a National Cancer Institute pilot project for the acceleration of translational research. *Clinical Cancer Research*.

[86] Bendle GM, Linnemann C, Hooijkaas AI (2010). Lethal graft-versus-host disease in mouse models of T cell receptor gene therapy. *Nature Medicine*.

[87] Ciceri F, Bonini C, Marktel S (2007). Antitumor effects of HSV-TK-engineered donor lymphocytes after allogeneic stem-cell transplantation. *Blood*.

[88] Tiberghien P, Ferrand C, Lioure B (2001). Administration of herpes simplex-thymidine kinase-expressing donor T cells with a T-cell-depleted allogeneic marrow graft. *Blood*.

[89] Ciceri F, Bonini C, Stanghellini MTL (2009). Infusion of suicide-gene-engineered donor lymphocytes after family haploidentical haemopoietic stem-cell transplantation for leukaemia (the TK007 trial): a non-randomised phase I-II study. *The Lancet Oncology*.

[90] Berger C, Flowers ME, Warren EH, Riddell SR (2006). Analysis of transgene-specific immune responses that limit the in vivo persistence of adoptively transferred HSV-TK-modified donor T cells after allogeneic hematopoietic cell transplantation. *Blood*.

[91] Straathof KC, Pulè MA, Yotnda P (2005). An inducible caspase 9 safety switch for T-cell therapy. *Blood*.

[92] Hacein-Bey-Abina S, Von Kalle C, Schmidt M (2003). LMO2-associated clonal T cell proliferation in two patients after gene therapy for SCID-X1. *Science*.

[93] Hacein-Bey-Abina S, Garrigue A, Wang GP (2008). Insertional oncogenesis in 4 patients after retrovirus-mediated gene therapy of SCID-X1. *Journal of Clinical Investigation*.

[94] Howe SJ, Mansour MR, Schwarzwaelder K (2008). Insertional mutagenesis combined with acquired somatic mutations causes leukemogenesis following gene therapy of SCID-X1 patients. *Journal of Clinical Investigation*.

[95] Davé UP, Jenkins NA, Copeland NG (2004). Gene therapy insertional mutagenesis insights. *Science*.

[96] Woods NB, Bottero V, Schmidt M, Von Kalle C, Verma IM (2006). Gene therapy: therapeutic gene causing lymphoma. *Nature*.

[97] Pike-Overzet K, de Ridder D, Weerkamp F (2007). Ectopic retroviral expression of LMO2, but not IL2R*γ*, blocks human T-cell development from CD34+ cells: implications for leukemogenesis in gene therapy. *Leukemia*.

[98] Modlich U, Schambach A, Brugman MH (2008). Leukemia induction after a single retroviral vector insertion in Evi1 or Prdm16. *Leukemia*.

[99] Yu SF, Von Ruden T, Kantoff PW (1986). Self-inactivating retroviral vectors designed for transfer of whole genes into mammalian cells. *Proceedings of the National Academy of Sciences of the United States of America*.

[100] Modlich U, Bohne J, Schmidt M (2006). Cell-culture assays reveal the importance of retroviral vector design for insertional genotoxicity. *Blood*.

[101] Montini E, Cesana D, Schmidt M (2009). The genotoxic potential of retroviral vectors is strongly modulated by vector design and integration site selection in a mouse model of HSC gene therapy. *Journal of Clinical Investigation*.

[102] Zychlinski D, Schambach A, Modlich U (2008). Physiological promoters reduce the genotoxic risk of integrating gene vectors. *Molecular Therapy*.

[103] Thornhill SI, Schambach A, Howe SJ (2008). Self-inactivating gammaretroviral vectors for gene therapy of x-linked severe combined immunodeficiency. *Molecular Therapy*.

[104] Bushman F, Lewinski M, Ciuffi A (2005). Genome-wide analysis of retroviral DNA integration. *Nature Reviews Microbiology*.

[105] Wu X, Li Y, Crise B, Burgess SM (2003). Transcription start regions in the human genome are favored targets for MLV integration. *Science*.

[106] Schröder ARW, Shinn P, Chen H, Berry C, Ecker JR, Bushman F (2002). HIV-1 integration in the human genome favors active genes and local hotspots. *Cell*.

[107] Mitchell RS, Beitzel BF, Schroder ARW (2004). Retroviral DNA integration: ASLV, HIV, and MLV show distinct target site preferences. *PLoS Biology*.

[108] Cattoglio C, Facchini G, Sartori D (2007). Hot spots of retroviral integration in human CD34+ hematopoietic cells. *Blood*.

[109] Recchia A, Bonini C, Magnani Z (2006). Retroviral vector integration deregulates gene expression but has no consequence on the biology and function of transplanted T cells. *Proceedings of the National Academy of Sciences of the United States of America*.

